# Effects of Provisional Cement Cleaning Methods on Resin–Dentin Bond Strength Following Immediate Dentin Sealing with Different Adhesive Systems

**DOI:** 10.3390/jfb17020098

**Published:** 2026-02-16

**Authors:** Zeynep Aydin, Cemile Kedici Alp, Osman F. Aydin

**Affiliations:** 1 Department of Restorative Dentistry, Faculty of Dentistry, Gazi University, Emek, Ankara 06490, Türkiye; zeyneporan@gazi.edu.tr; 2 Department of Restorative Dentistry, Faculty of Dentistry, Ankara Medipol University, Çankaya, Ankara 06570, Türkiye; osman.aydin@ankaramedipol.edu.tr

**Keywords:** dentin sealing, provisional cement, adhesive, shear bond strength, surface cleaning

## Abstract

This study evaluated the effects of different provisional luting cement removal methods on the shear bond strength (SBS) of resin cement to dentin following immediate dentin sealing (IDS) performed with two adhesive systems. A total of 168 extracted, caries-free human third molars were used, of which 144 were allocated for SBS testing and 24 for scanning electron microscopy (SEM) analysis. Specimens were assigned according to the IDS protocol (no IDS, IDS with OptiBond FL, or IDS with G2-Bond), followed by provisional cementation using an eugenol-free temporary cement. Contaminated surfaces were subsequently cleaned with a hand scaler, aluminum oxide (Al_2_O_3_) air abrasion, or Katana Cleaner prior to final bonding with a dual-cure resin cement. SBS was measured after 24 h of water storage, and surface morphology was evaluated by SEM at 2500× magnification. IDS significantly increased SBS under uncontaminated conditions, with G2-Bond-based IDS exhibiting higher bond strength values than specimens without IDS. However, provisional cement contamination significantly reduced SBS regardless of the cleaning method applied, and none of the tested protocols fully restored the bond strength observed in uncontaminated IDS-treated dentin. SEM analysis revealed residual cement remnants and surface alterations after cleaning, even in specimens that appeared macroscopically clean. Within the limitations of this in vitro study, IDS enhances resin–dentin bonding when contamination is avoided; however, current mechanical and chemical cleaning methods are insufficient to completely recover bond strength compromised by provisional cement contamination, highlighting the importance of preventing contamination and preserving IDS layer integrity during indirect restorative procedures.

## 1. Introduction

With advances in adhesive technology and prosthetic/restorative materials, the demand for indirect restorations has increased owing to their superior esthetic and mechanical properties compared with direct restorations. Tooth preparation for indirect restorations often exposes dentin to varying degrees [1]. Temporary restorations are routinely placed to protect the prepared tooth structure and maintain laboratory workflow during the provisional phase [2]. Following fabrication of the definitive indirect restoration, the provisional restoration is removed, an adhesive is applied to the dentin substrate, and resin luting cement is used for final bonding [3]. In clinical practice, this sequence often leads to bonding procedures performed on contaminated dentin surfaces, which may compromise hybrid layer formation and reduce bond strength [4]. To overcome this limitation, the Immediate Dentin Sealing (IDS) technique has been proposed [5]. IDS involves the application of an adhesive system immediately after tooth preparation, enabling hybridization of freshly cut, uncontaminated dentin prior to provisionalization [6]. This approach provides bonding to a clean dentin surface and has been shown to reduce bacterial penetration and postoperative dentin sensitivity during the provisional phase [7,8].

In principle, IDS may be performed using various adhesive systems; however, two-step self-etch or three-step etch-and-rinse systems are generally recommended [4,9]. Despite ongoing advances in adhesive dentistry, uncertainty persists about the performance of contemporary adhesive strategies (such as total-etch, self-etch, and universal systems) in the context of IDS. Previous investigations have suggested that conventional three-step etch-and-rinse adhesives represent a reliable option for long-term IDS performance [9]. Carvalho et al. [10] reported that OptiBond FL is particularly suitable for IDS owing to its elastic modulus and high filler content, which contribute to improved stress distribution at the resin–dentin interface. Scanning electron microscopy (SEM) observations from that study demonstrated a stable, mechanically resistant adhesive interface even after provisionalization and surface-cleaning procedures, supporting its use in IDS protocols without the need for an additional flowable resin layer.

Indirect restorations are fabricated extraorally, and clinical procedures frequently require provisionalization following impression making in the initial appointment [3]. Inadequate removal of temporary luting cement may result in contamination of the dentin surface, leading to reduced surface free energy and compromised adhesion [11]. Beyond surface energy considerations, the chemical composition of the temporary luting cement is also critical. Eugenol-containing cements have been shown to interfere with resin polymerization and adversely affect the physical properties and bond strength of resin cements. Nevertheless, several studies indicate that even eugenol-free temporary cements may negatively influence resin–dentin bonding. Residual cement deposits on the dentin surface can hinder the interaction between acidic functional monomers and dentin’s inorganic components, thereby impairing adhesive performance [12].

While IDS effectively limits contamination from impression materials and provisional cements, the integrity of the IDS layer itself may be compromised during removal of temporary restorations prior to definitive cementation. Thorough elimination of temporary cement residues is therefore essential to preserve dentin–adhesive contact and optimize bond strength [13,14]. However, mechanical cleaning methods alone may be insufficient, as microscopic cement remnants can persist on dentin surfaces that appear macroscopically clean, leading to reduced bonding effectiveness [14]. To address this limitation, chemical cleaning agents have been introduced. Katana Cleaner, a moderately acidic (pH 4.5) solution containing 10-methacryloyloxydecyl dihydrogen phosphate (MDP) and triethanolamine, has been developed to chemically remove organic contaminants from dental substrates and restorations [15]. Despite these advancements, consensus has not been reached regarding the most effective cleaning protocol for dentin surfaces that have undergone IDS and subsequent provisional cementation.

Therefore, the present study aimed to evaluate how different temporary luting cement removal protocols functionally modify the dentin surface and influence the bonding performance of dual-cure resin cement following IDS performed with two adhesive systems. Shear bond strength testing and scanning electron microscopy were used to correlate mechanical outcomes with surface-level morphological changes. From a biomaterials perspective, this investigation focuses on the functional behavior of resin–dentin adhesive interfaces and surface material interactions under clinically relevant contamination and cleaning conditions. The null hypotheses tested were: (1) IDS performed with different adhesive systems does not affect the shear bond strength of dual-cure resin cement to dentin; (2) temporary cement cleaning methods do not influence shear bond strength; and (3) IDS and cleaning procedures do not induce discernible differences in dentin surface morphology as assessed by SEM.

## 2. Materials and Methods

A complete list of the materials used for adhesive procedures, provisional cementation, and final luting is presented in Table 1.

**Figure 1:**
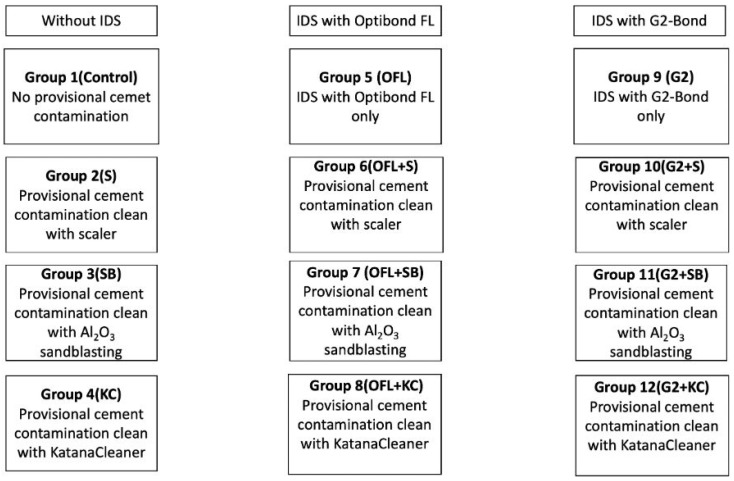
This flow chart shows the groups of the study. The first row of each column represents the main experimental groups, while the boxes below indicate the corresponding subgroups derived from each main group according to the applied provisional cement cleaning protocols. IDS: immediate dentin sealing; OFL: OptiBond FL; G2: G2-Bond; S: hand scaler; SB: aluminum oxide air abrasion (sandblasting); KC: Katana Cleaner.

This in vitro study was approved by the Ethics Committee of Gazi University Faculty of Dentistry. A total of 168 extracted, caries-free human third molars were collected, of which 144 specimens were allocated for shear bond strength (SBS) testing and 24 specimens for SEM analysis. An a priori power analysis was conducted for a two-way ANOVA targeting the interaction between adhesive type and cleansing method. The analysis indicated that, to detect a medium-to-large effect size (Cohen’s f = 0.40) with 90% statistical power at a significance level of α = 0.05, a minimum total sample size of 116 observations was required. Given that the experimental design comprised 12 subgroups, 12 specimens were allocated to each subgroup, for a total sample size of 144. The anticipated effect size (f = 0.40) was defined a priori based on clinical considerations, reflecting the magnitude of differences considered relevant for in vitro resin–dentin bond strength comparisons within a balanced multi-group experimental design. Following extraction, residual soft tissues were removed, and the teeth were embedded in PVC molds using autopolymerizing acrylic resin (IMICRYL, Konya, Turkey), leaving the occlusal surfaces exposed. Flat superficial dentin surfaces were obtained by removing the occlusal enamel using a low-speed diamond saw (Isomet 1000, Buehler, Lake Bluff, IL, USA) under continuous water cooling. The dentin surfaces were subsequently standardized by sequential polishing with 600-, 800-, and 1000-grit silicon carbide abrasive papers under water for 10 s each, followed by ultrasonic cleaning in distilled water for 5 min. The 144 specimens allocated for SBS testing were randomly assigned to 12 experimental groups according to the applied IDS protocol and subsequent surface treatment. Groups 1–4 served as non-sealed controls, in which no immediate dentin sealing (IDS) was performed. IDS was applied in Groups 5–8 using OptiBond FL and in Groups 9–12 using G2-Bond, followed by provisional cementation and surface cleaning procedures according to the respective group assignments. The study groups are depicted in Figure 1.

### 2.1. IDS Procedures

IDS procedures were carried out strictly in accordance with the manufacturer’s instructions. For OptiBond FL (Kerr, Orange, CA, USA), dentin surfaces were etched with 37% phosphoric acid for 15 s, rinsed with water for 15 s, and gently air-dried for 5 s. The primer was applied for 15 s, air-dried for 5 s, and then the adhesive resin was applied for 15 s. The adhesive layer was then air-thinned for 3 s and light-cured for 30 s using an LED curing unit (D-Light Pro, GC, Tokyo, Japan). The light output intensity was verified using a radiometer and confirmed to be 1200 mW/cm^2^.

For G2-Bond (GC, Tokyo, Japan), the primer was applied for 10 s and air-blown for 5 s. The bonding agent was subsequently applied, air-thinned for 5 s, and light-cured for 30 s using the same curing unit and irradiation parameters. Groups 1, 5, and 9 served as uncontaminated controls. In the remaining groups, an eugenol-free provisional cement (TempBond NE, Kerr, Orange, CA, USA) was applied at a standardized thickness of 2.0 mm. A constant load of 500 g was applied for 60 s using a glass slab, after which the specimens were stored in distilled water for 24 h.

### 2.2. Surface Treatments for Removal of Provisional Cement

Surface cleaning protocols were applied exclusively to provisional cement contaminated specimens (Groups 2–4, 6–8, and 10–12), whereas Groups 1, 5, and 9 served as uncontaminated controls. Temporary cement removal was performed according to the assigned subgroup:

*Subgroup S* (*Scaler*, *n* = 36)*:* Provisional cement was removed using a hand scaler with closely spaced, overlapping strokes under moderate pressure until the dentin surface appeared macroscopically clean and free of visible residues. All manual cleaning procedures were performed by a single calibrated operator under standardized illumination, and the endpoint was defined as the absence of visible cement remnants upon visual inspection.

*Subgroup SB* (*Sandblasting*, *n* = 36)*:* Dentin surfaces were treated with 50 μm aluminum oxide (Al_2_O_3_) particles for 20 s at 2.8 bar pressure, from a distance of 10 mm, using a rotating motion (Dento-Prep, Rønvig Dental, Denmark).

*Subgroup KC* (*Katana Cleaner*, *n* = 36)*:* Katana Cleaner (Kuraray Noritake Dental Inc., Okayama, Japan) was applied to the surface with a microbrush for 10 s and thoroughly rinsed with water until the characteristic purple coloration disappeared.

### 2.3. Luting Procedures

Following surface cleaning, tooth primer (Panavia V5 tooth primer, Kuraray Noritake Dental Inc., Tokyo, Japan) was applied to the dentin surfaces in accordance with the manufacturer’s instructions. Polyethylene molds (2 mm in diameter × 2 mm in height) were then positioned on the dentin surfaces and filled with a dual-curing resin cement (Panavia V5, Kuraray Noritake Dental Inc., Okayama, Japan). The resin cement was light-cured for 40 s using the same LED curing unit. After polymerization, the molds were carefully removed using a #11 scalpel blade. The specimens were subsequently stored in distilled water at 37 °C for 24 h prior to testing.

### 2.4. Shear Bond Strength Test

All shear bond strength measurements were performed after 24 h of water storage, reflecting early bond strength performance. Shear bond strength (SBS) testing was conducted using a universal testing machine (Shimadzu AG-IS Autograph, Kyoto, Japan) at a crosshead speed of 1 mm/min. Bond strength values (MPa) were calculated by dividing the maximum load at failure (N) by the bonded surface area (mm^2^).

### 2.5. Examination of Surface Topography

For SEM evaluation, two additional specimens were prepared for each experimental group and examined using a field-emission scanning electron microscope (FE-SEM) (Hitachi SU5000, Tokyo, Japan). These specimens were processed according to the respective IDS and cleaning protocols but were not subjected to resin-cement bonding. After drying for 24 h, specimens were gold-sputtered (Leica ACE 200, Vienna, Austria) and observed at 2500× magnification to evaluate dentin or IDS surface morphology following the cleaning procedures.

### 2.6. Failure Mode Types

Following bond-strength testing, fractured specimens were examined at 100× magnification using a stereomicroscope (SZH-131, Olympus, Tokyo, Japan). Failure modes were classified in accordance with ISO 10365:2022 [16] as follows:

- AF: Adhesive failure at the dentin–adhesive interface
- CSF: Cohesive failure within dentin
- CF: Cohesive failure within cement
- ACFP: Mixed adhesive and cohesive failure

### 2.7. Statistical Analysis

An *a priori* power analysis was conducted for a two-way ANOVA targeting the interaction between adhesive type and cleansing method. The analysis indicated that, to detect a medium-to-large effect size (Cohen’s f = 0.40) with 90% statistical power at a significance level of α = 0.05, a minimum total sample size of 116 observations was required. Given that the experimental design comprised 12 subgroups, 12 specimens were allocated to each subgroup, for a total sample size of 144. The anticipated effect size (f = 0.40) was defined a priori based on clinical considerations, reflecting the magnitude of differences considered relevant for in vitro resin–dentin bond strength comparisons within a balanced multi-group experimental design. All power calculations were performed using G*Power software (version 3.1.9.6; Franz Faul, Universität Kiel, Kiel, Germany). The distribution of shear bond strength (SBS) data was assessed for normality using the Kolmogorov–Smirnov test, and homogeneity of variances was evaluated using Levene’s test. As the assumptions for parametric analysis were not met, non-parametric statistical methods were applied. SBS values were therefore summarized using median and interquartile range (25th–75th percentiles).

Within each cleansing method, differences in SBS among adhesive protocols, as well as within each adhesive protocol, were analyzed using the Kruskal–Wallis test. When a significant omnibus effect was detected, post hoc pairwise comparisons were performed using Dunn’s test with Bonferroni correction to identify between-group differences. Since failure modes were categorical data, comparisons of failure type distributions were performed using Pearson’s χ^2^ test. In cases where expected cell counts were insufficient due to the number of subgroups and failure categories, the Fisher Freeman Halton exact test was applied. These analyses were conducted to provide an exploratory overview of failure pattern distributions across experimental groups. All statistical analyses were carried out using IBM SPSS Statistics (version 25; IBM Corp., Armonk, NY, USA). Unless otherwise stated, a *p*-value < 0.05 was considered statistically significant. Bonferroni correction was applied to control the type I error rate associated with multiple comparisons.

## 3. Results

### 3.1. Shear Bond Strength

In specimens bonded without immediate dentin sealing (IDS), shear bond strength (SBS) values did not differ significantly among the evaluated cleaning methods (*p* = 0.120). In contrast, when IDS was performed using OptiBond FL (OFL), significant differences in SBS were observed among the cleaning protocols (*p* = 0.004). Specifically, the Al_2_O_3_ air-abrasion subgroup exhibited significantly lower SBS values than those of uncontaminated specimens (*p* = 0.014).

Similarly, specimens treated with G2-Bond-based IDS showed significant differences in SBS across cleaning methods (*p* < 0.001). Compared with uncontaminated specimens, SBS values were significantly reduced following manual cleaning, Al_2_O_3_ air-abrasion, and Katana Cleaner application (*p* < 0.001, *p* < 0.001, and *p* = 0.005, respectively) (Table 2).

When only uncontaminated specimens were analyzed, SBS values differed significantly among adhesive protocols (*p* < 0.001). This difference was primarily associated with higher SBS values recorded for G2-Bond-based IDS compared with the no-IDS protocol (*p* < 0.001). However, differences between the no-IDS and OFL-based IDS groups, as well as between OFL- and G2-Bond-based IDS, were not statistically significant after Bonferroni correction (both *p* = 0.030).

**Figure 2:**
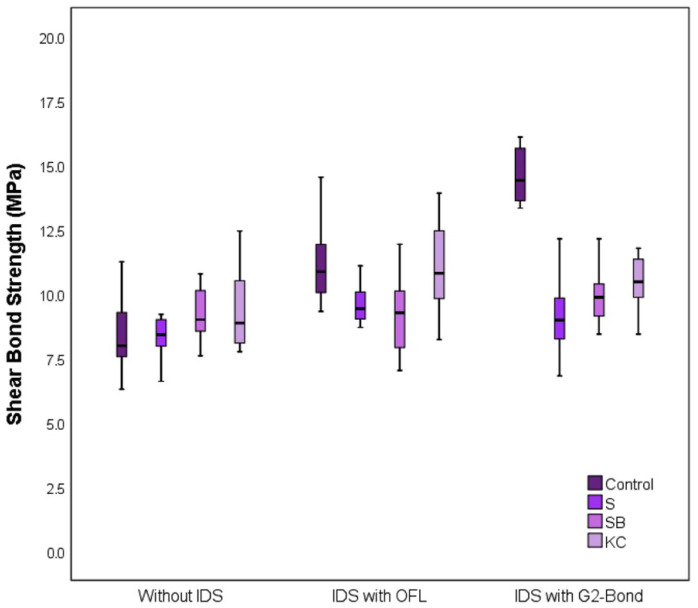
Box and whisker plot graph for Shear Bond Strength (SBS) levels in terms of adhesive types and cleansing methods. The horizontal dark lines in the middle of each box indicate the median, while the top and bottom borders of the box mark the 25th and 75th percentiles, respectively. The whiskers above and below the box mark the maximum and minimum values for SBS.

Among contaminated specimens, no significant differences in SBS were observed between adhesive protocols following manual cleaning (*p* = 0.081), Al_2_O_3_ air-abrasion (*p* = 0.428), or Katana Cleaner application (*p* = 0.056). Box-and-whisker plots illustrating SBS values according to adhesive protocol and cleaning method are presented in Figure 2.

### 3.2. Failure Mode Analysis

**Figure 3:**
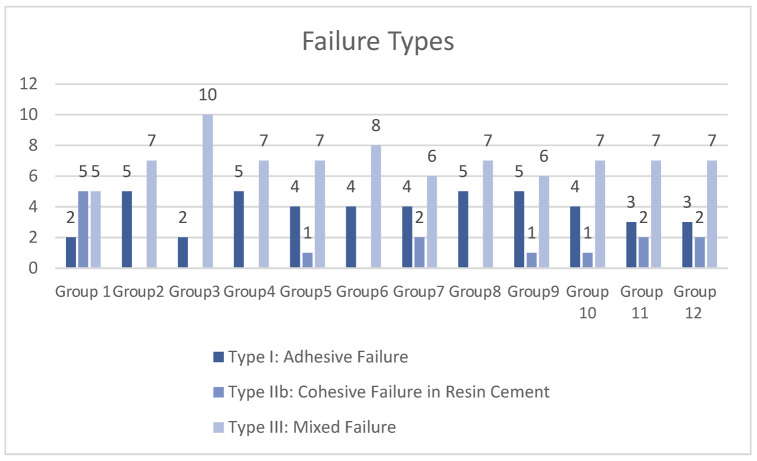
Distribution of failure types observed after shear bond strength testing. *X*-axis: experimental groups (Groups 1–12; corresponding to the different adhesive/cleaning combinations). *Y*-axis: number of specimens (0–12). Each bar represents the number of specimens within the respective group that exhibited the corresponding failure mode.

The distribution of failure modes for each experimental group is presented in Figure 3. Across all groups, mixed failures were the most frequently observed failure pattern, whereas cohesive failures occurred least frequently. The distribution of failure modes according to adhesive protocol and cleansing method is presented in Table 3. Failure patterns were predominantly classified as Type III (mixed failure) across all experimental groups, whereas Type IIb (cohesive failure within resin cement) occurred less frequently.

When comparisons were made among cleansing methods within each adhesive protocol, no statistically significant differences were detected in the distribution of failure types for the IDS with OFL and IDS with G2-Bond groups (*p* > 0.05). In the Without IDS group, a significant difference was observed among cleansing methods for Type IIb failures, with the control subgroup exhibiting a higher frequency compared with the mechanically cleaned subgroups (*p* = 0.037).

Comparisons among adhesive protocols within each cleansing method did not reveal statistically significant differences in failure mode distributions (*p* > 0.05). Overall, failure mode patterns were largely comparable across adhesive systems and cleaning protocols, and no consistent association between a specific experimental condition and a distinct failure type was identified.

### 3.3. SEM Analysis of the Specimens

**Figure 4:**
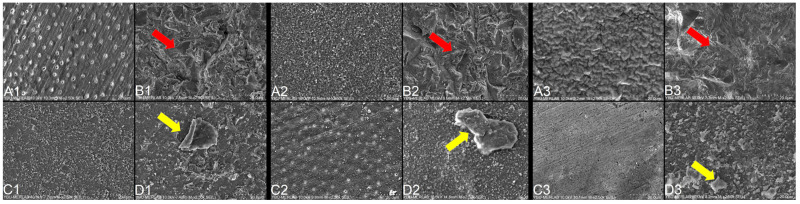
Representative SEM images (2500× magnification) of dentin surfaces after different temporary cement cleaning methods, with or without immediate dentin sealing (IDS). Without IDS: (**A1**) Dentin surface without IDS and without contamination; (**B1**) Temporary cement removed using 50 μm Al_2_O_3_ sandblasting; (**C1**) Temporary cement removed using Katana Cleaner; (**D1**) Temporary cement manually removed. With IDS using OptiBond FL: (**A2**) Dentin surface sealed with OptiBond FL, no contamination; (**B2**) Temporary cement removed using 50 μm Al_2_O_3_ sandblasting; (**C2**) Temporary cement removed using Katana Cleaner; (**D2**) Temporary cement manually removed. With IDS using G2-Bond: (**A3**) Dentin surface sealed with G2-Bond, no contamination; (**B3**) Temporary cement removed using 50 μm Al_2_O_3_ sandblasting; (**C3**) Temporary cement removed using Katana Cleaner; (**D3**) Temporary cement manually removed. Notes: Red arrows indicate multiple grooves formed as a result of alumina particle impact during air-abrasion. Yellow arrows highlight residual temporary cement remnants remaining on the surface after mechanical cleaning with a scaler.

Representative SEM images of dentin and IDS-treated surfaces obtained at 2500× magnification are shown in Figure 4. SEM analysis demonstrated the presence of residual temporary cement particles on dentin surfaces following all cleaning procedures, including specimens that exhibited relatively high SBS values.

In the control group without IDS, SEM images revealed open dentinal tubule orifices. Following IDS with OptiBond FL (Figure 4(A2)), filler particles originating from the adhesive layer were visible on the surface. In contrast, IDS performed with G2-Bond (Figure 4(A3)) resulted in a relatively thicker adhesive layer covering the dentin surface, oriented in the direction of air thinning.

Specimens cleaned using Al_2_O_3_ air-abrasion exhibited roughened surfaces characterized by pits and microcracks (Figure 4(B1–B3)). These surface alterations appeared more superficial in Group 10, in which IDS was performed using G2-Bond. Katana Cleaner-treated specimens (Figure 4(C1–C3)) showed small fragmented remnants of temporary cement on the surface; however, the IDS layer appeared largely preserved in Groups 7 and 11.

In specimens where provisional cement was removed using hand instruments (Figure 4(D1–D3)), larger residual cement fragments were evident. Such remnants may have interfered with both micromechanical retention and chemical interaction between the resin cement and the underlying dentin surface.

## 4. Discussion

Immediate dentin sealing (IDS) has been widely advocated as a strategy to enhance the bonding performance of indirect restorations by enabling adhesive application on freshly prepared, uncontaminated dentin surfaces [17,18]. While the general benefits of IDS have been well documented, the present study provides additional insight into how different adhesive systems and post-provisional cleaning protocols interact with a pre-polymerized IDS layer. Accordingly, the first null hypothesis was rejected because IDS significantly increased SBS under uncontaminated conditions; the second null hypothesis was rejected because provisional cement contamination significantly reduced SBS irrespective of the cleaning protocol; whereas the third null hypothesis was not fully rejected, as none of the cleaning methods was able to restore SBS values to those observed under uncontaminated IDS conditions.

Both OptiBond FL- and G2-Bond-based IDS protocols resulted in higher SBS values than non-IDS controls, supporting previous evidence that pre-hybridization of dentin can improve resin–dentin interaction [9,10,18]. However, the present results extend existing knowledge by demonstrating that the magnitude of this benefit is strongly influenced by adhesive composition when contamination is avoided. In uncontaminated conditions, G2-Bond exhibited numerically higher SBS values than OptiBond FL, suggesting that factors beyond filler content alone contribute to IDS effectiveness. This observation highlights adhesive chemistry as a critical determinant of IDS performance rather than merely mechanical reinforcement by filler loading.

The comparatively favorable performance of G2-Bond can be attributed to its HEMA-free formulation and Dual-H technology. Dual h technology can promote a transition from hydrophilic behavior during dentin infiltration to a more hydrophobic polymer network after curing [19]. These characteristics may reduce water sorption and hydrolytic degradation within the adhesive layer. By contrast, HEMA-containing systems such as OptiBond FL, while effective in facilitating dentin wetting and infiltration, are known to be more susceptible to water uptake and long-term degradation [20,21]. It should be emphasized, however, that these interpretations remain inferential, as direct evaluation of water sorption or polymer stability was beyond the scope of the present study.

Functional monomer chemistry provides further insight into the observed adhesive behavior. According to the adhesion-decalcification (A-D) concept, durable adhesion depends on stable chemical interaction with residual hydroxyapatite rather than excessive demineralization [22]. G2-Bond contains 10-MDP, which is capable of forming stable MDP-Ca nanolayers that can enhance the chemical durability and nanomechanical integrity of the hybrid layer [23]. In contrast, OptiBond FL primarily relies on GPDM, an early phosphate monomer that promotes demineralization but forms less stable calcium complexes, potentially resulting in interfacial phases that are more prone to degradation over time [24]. In addition, the coexistence of 4-MET in the G2-Bond primer and the use of acetone as a solvent may facilitate monomer diffusion and solvent evaporation, contributing to a more homogeneous adhesive layer [25,26,27,28,29]. Together, these chemical characteristics may partly explain the adhesive-dependent differences observed under uncontaminated IDS conditions.

Despite these material-related advantages, provisional cementation emerged as a critical factor compromising the effectiveness of IDS. Consistent with previous reports, contamination associated with temporary cements negatively affected resin–dentin bonding, even when non-eugenol formulations were used [11,12,13]. Residual cement particles may reduce surface free energy and obstruct chemical interaction between acidic functional monomers and the dentin substrate [12,13], thereby limiting the functional contribution of the IDS layer. In the present study, all provisionalized groups demonstrated reduced SBS values compared with uncontaminated IDS specimens, regardless of the cleaning protocol used, underscoring IDS’s sensitivity to contamination-related alterations.

The influence of cleaning procedures became particularly apparent when an IDS layer was present. While cleaning methods did not significantly affect SBS on non-sealed dentin, all post-provisional cleaning approaches resulted in reduced bond strength for both IDS systems. This finding suggests that a pre-polymerized IDS layer is more vulnerable to mechanical and physicochemical intervention than bare dentin. Previous studies have shown that mechanical cleaning methods, such as hand instrumentation and aluminum oxide air abrasion, may fail to completely eliminate residual cement and may also thin or disrupt the IDS layer itself [30,31,32,33,34,35]. In particular, air abrasion has been associated with removal of the oxygen-inhibited surface and localized exposure of underlying adhesive or dentin, potentially compromising interfacial continuity and functional bonding potential [31,34]. However, it should be emphasized that the negative effects observed following air abrasion may be protocol dependent. The relatively aggressive air-abrasion parameters used in the present study (50 μm particles, 2.8 bar pressure, 20 s application) may have contributed not only to incomplete removal of provisional cement remnants but also to iatrogenic disruption or thinning of the pre-polymerized IDS layer. Özcan et al. [31] reported that SEM analysis revealed increased surface roughness, particularly in the air-abraded groups, which may have influenced the wettability of the IDS-treated surfaces. Therefore, the reduced bond strength observed after air abrasion should be interpreted as a combined effect of residual contamination and mechanical damage to the IDS layer, rather than as a universal limitation of air-abrasion cleaning.

Katana Cleaner was developed to improve decontamination by combining the surfactant action of 10-MDP and triethanolamine [36,37,38]. Nevertheless, its application did not restore bond strength to levels observed in uncontaminated IDS specimens. SEM observations showed the presence of cement residues on the treated surfaces. The SEM findings in this study should be interpreted as qualitative support rather than direct proof of causality. The use of Panavia V5, a dual-cure resin cement containing 10-MDP and employing a “touch-and-cure” mechanism, may also have reduced the sensitivity of SBS outcomes to surface cleanliness by promoting chemical interaction at the resin–dentin interface [39], potentially masking differences between cleaning protocols.

Failure mode analysis further supported these observations, with mixed and adhesive failures predominating across all groups, indicating that fractures commonly occurred at or near the adhesive interface. This pattern is consistent with previous studies on IDS-treated dentin following provisional cement removal [32]. SEM analyzes revealed microscopic remnants that were not detectable by naked-eye inspection; However, in the present study, SEM observations were intended to provide representative and qualitative morphological support to the mechanical findings and should not be interpreted as direct quantitative or causal evidence of adhesive performance. Future studies employing image-processing techniques that enable semi-quantitative analyzes such as calculating the proportion of residual material within a defined area of interest may further strengthen the interpretation of surface characteristics.

From a clinical perspective, the present findings suggest that although IDS can enhance resin–dentin bonding under short-term, non-aged conditions, its effectiveness is highly dependent on strict control of contamination during the provisional phase. Aggressive mechanical cleaning strategies may negate the benefits of IDS by compromising the integrity of the adhesive layer. Importantly, none of the evaluated cleaning methods fully restored the bond strength achieved under uncontaminated IDS conditions, highlighting the limitations of current decontamination strategies.

Several bond strength testing methodologies have been described in the literature, including shear and tensile tests performed at different specimen dimensions. Among these, shear bond strength (SBS) testing remains one of the most widely applied methods in adhesive dentistry, particularly in studies aimed at comparing multiple surface treatment protocols and adhesive strategies. In the present study, SBS testing was deliberately selected to enable standardized and reproducible comparison of the relative effects of different provisional cement cleaning methods and adhesive systems. Although SBS testing is associated with non-uniform stress distribution and lower sensitivity than microtensile testing, it is well suited for identifying comparative trends among experimental groups rather than determining absolute bond strength values [31]. In line with this approach, SBS testing has also been used in previous studies conducted by Özdoğan et al. [40] and Iwama et al. [41] to evaluate the effect of resin cement on dentin bond performance, thereby supporting the suitability of this method for comparative experimental designs.

Several limitations of this study should be acknowledged. In the present study, shear bond strength was evaluated at an early stage, without the application of artificial aging protocols (thermal or mechanical) or long-term storage in artificial saliva. Therefore, future studies incorporating artificial aging procedures, alternative provisional materials, and different bond strength testing methods are warranted to further clarify the long-term functional performance of IDS following provisional cement contamination.

## 5. Conclusions

Within the limitations of this in vitro study, the following conclusions can be drawn:

1. Immediate dentin sealing (IDS) enhances resin–dentin shear bond strength when contamination is avoided.
2. IDS performed with G2-Bond yielded comparable or numerically higher bond strength values than IDS performed with OptiBond FL.
3. Provisional cement contamination adversely affected bond strength, irrespective of the cleaning protocol applied.
4. None of the evaluated cleaning methods fully restored the bond strength observed under uncontaminated IDS conditions.
5. SEM observations indicated the presence of residual cement remnants and surface alterations even after cleaning procedures.

From a clinical perspective, the cleaning methods applied may not have completely removed the temporarily placed cement from the dentin surface. Given the limitations of the current study, future research is needed to more comprehensively evaluate the effects of temporarily cemented materials on the entire dentin surface.

## Figures and Tables

**Table 1:** The materials and composition used in the study.

Manufacturer (LOT Number)	Material	Composition According to Manufacturer
OptiBond FL, Kerr Corporation, Orange, CA, USA (9558965)	Three-step etch-and-rinse adhesive	(Primer): HEMA, GPDM, PAMM, ethanol, water, photoinitiator. (Adhesive): TEGDMA, UDMA, GPDM, HEMA, BİS-GMA, filler, photoinitiator
G2-Bond, GC Corporation, Tokyo, Japan (2210071)	Two-step Universal adhesive	(Primer): 4-MET, 10-MDP, 10-MDTP, Dimethacrylate monomer, acetone, water, initiators, filler. (Adhesive): Dimethacrylate monomer, Bis-GMA, filler, photoinitiator
Temp Bond Ne, Kerr Corporation, Orange, CA, USA (8485854)	Eugenol-free provisional cement	Accelerator: Orthoethoxybenzoic acid, carnauba wax, octanoic acid Base: Zinc oxide (Zno—85–90%), mineral oil
Katana Cleaner, Kuraray Noritake Dental Inc., Okayama, Japan (9C0034)	Chemical cleaner	Water, 10-MDP, triethanolamine, polyethylene glycol, stabilizer and dyes. (pH = 4.5)
Dento-Prep, Ronvig Dental, Daugaard, Denmark (230755347)	Air-abrasion Sandblaster	
Panavia V5, Kuraray Noritake Dental Inc., Okayama, Japan (3V0234)	Dual-cure resin cement	Paste A: Bis-GMA, TEGDMA, hydrophobic aromatic dimethacrylate, hydrophilic aliphatic dimethacrylate, initiators, accelerators, silanized barium glass filler, silanized fluoroaluminosilicate glass filler, colloidal silica. Paste B: Bis-GMA, hydrophobic aromatic dimethacrylate, hydrophilic aliphatic dimethacrylate, silanized barium glass filler, silanized aluminum oxide filler, accelerators, di-camphorquinone, pigments. (Primer): MDP, HEMA, hydrophilic aliphatic dimethacrylate, accelerator, water (pH = 2.0).

**Table 2:** Shear (SBS) bond strengths of dual-cure resin cement on dentin after cleaning methods.

	Without IDS	IDS with OFL	IDS with G2-Bond	*p*-Value ^1,3^
Control	7.99 (7.50–9.45) ^A^	10.86 (10.05–12.08) ^A,B,a^	14.40 (13.55–15.82) ^B,a^	<0.001
S	8.41 (7.89–9.11)	9.43 (8.94–10.22) ^a,b^	8.98 (8.04–9.97) ^b^	0.081
SB	9.01 (8.49–10.22)	9.27 (7.58–10.15) ^b^	9.87 (9.07–10.56) ^b^	0.428
KC	8.87 (8.06–10.62)	10.80 (9.65–12.55) ^a,b^	10.47 (9.72–11.48) ^b^	0.056
*p*-value ^2,3^	0.120	0.004	<0.001	

Data were displayed as median (25th–75th) percentiles. ^1^ The comparisons among adhesive types within each cleansing method, according to the Bonferroni correction *p* < 0.0125, were considered statistically significant. ^2^ The comparisons among cleansing methods within each adhesive type, according to the Bonferroni correction *p* < 0.0167, were considered statistically significant. ^3^ Kruskal–Wallis test. Adhesive types sharing the same uppercase letter were not significantly different (*p* > 0.0125). Cleansing methods sharing the same lowercase letter were not significantly different (*p* > 0.0167).

**Table 3:** Failure type distribution of dual-cure resin cement on dentin after cleansing methods.

	Without IDS	IDS with OFL	IDS with G2-Bond	*p*-Value ^1^
Type I				
Control	2 (16.7%)	4 (33.3%)	5 (41.7%)	0.400 ^3^
S	5 (41.7%)	4 (33.3%)	4 (33.3%)	>0.999 ^4^
SB	2 (16.7%)	4 (33.3%)	3 (25.0%)	0.887 ^4^
KC	5 (41.7%)	5 (41.7%)	3 (25.0%)	0.748 ^4^
*p*-value ^2^	0.304 ^3^	>0.999 ^4^	0.902 ^4^	
Type IIb				
Control	5 (41.7%) ^a^	1 (8.3%)	1 (8.3%)	0.165 ^4^
S	0 (0.0%) ^b^	0 (0.0%)	1 (8.3%)	>0.999 ^4^
SB	0 (0.0%) ^b^	2 (16.7%)	2 (16.7%)	0.516 ^4^
KC	0 (0.0%) ^b^	0 (0.0%)	2 (16.7%)	0.314 ^4^
*p*-value ^2^	0.002 ^4^	0.600 ^4^	>0.999 ^4^	
Type III				
Control	5 (41.7%)	7 (58.3%)	6 (50.0%)	0.913 ^3^
S	7 (58.3%)	8 (66.7%)	7 (58.3%)	0.890 ^3^
SB	10 (83.3%)	6 (50.0%)	7 (58.3%)	0.308 ^4^
KC	7 (58.3%)	7 (58.3%)	7 (58.3%)	>0.999 ^4^
*p*-value ^2^	0.217 ^3^	0.976 ^4^	0.968 ^3^	

^1^ The comparisons among adhesive types within each cleansing method, ^2^ The comparisons among cleansing methods within each adhesive type. ^3^ Pearson’s χ^2^ test, ^4^ Fisher Freeman Halton test. Among cleansing methods in the Without IDS group, those sharing the same lowercase letter did not differ significantly (*p* > 0.05).

## Data Availability

The original contributions presented in this study are included in the article. Further inquiries can be directed to the corresponding author.
